# Does physical activity benefit motor performance and learning of upper extremity tasks in older adults? – A systematic review

**DOI:** 10.1186/s11556-017-0181-7

**Published:** 2017-09-12

**Authors:** Lena Hübner, Claudia Voelcker-Rehage

**Affiliations:** Sports Psychology, Institute of Human Movement Science and Health, Faculty of Behavioural and Social Sciences, Chemnitz University of Technology, Thueringer Weg 11, 09126 Chemnitz, Germany

**Keywords:** Physical activity, Cardiovascular exercise, Acute exercise, Coordinative exercise, Aging, Motor performance, Motor learning

## Abstract

Upper extremity motor performance declines with increasing age. However, older adults need to maintain, learn new and relearn known motor tasks. Research with young adults indicated that regular and acute physical activity might facilitate motor performance and motor learning processes. Therefore, this review aimed to examine the association between chronic physical activity and acute bouts of exercise on motor performance and motor learning in upper extremity motor tasks in older adults. Literature was searched via Cochrane library, PubMED, PsycINFO and Scopus and 27 studies met all inclusion criteria. All studies dealt with the influence of chronic physical activity on motor performance or motor learning, no appropriate study examining the influence of an acute bout of exercise in older adults was found. Results concerning the association of chronic physical activity and motor performance are mixed and seem to be influenced by the study design, kind of exercise, motor task, and exercise intensity. Regarding motor learning, a high physical activity or cardiovascular fitness level seems to boost the initial phase of motor learning; results differ with respect to motor retention. Overall, (motor-coordinative) intervention studies seem to be more promising than cross-sectional studies.

## Background

Upper extremity motor performance as required for grasping, reaching or holding an object, declines with increasing age, having a strong impact on older adults’ activities of daily living ([13] for a review). Physical activity does not only have a positive influence on physiological health, psychological well-being, and cognitive performance [20, 26], it might also improve motor performance and motor learning processes. Up to now, most research on physical activity and motor performance or learning has been done with young adults revealing heterogeneous, but promising results ([28] for a review on motor performance in children and adolescents; [78] for a general review on motor learning). This positive association might also exist in older adults and might foster them to maintain performance levels, learn new and relearn known motor tasks as part of new task training, recreational pursuits, or rehabilitation. A positive association between physical activity and motor performance or learning in older adults would also offer promising areas of research, e.g., in a clinical setting for rehabilitation after stroke ([70] for a review).

### Some defining characteristics

When dealing with the effects of physical activity in motor performance and learning studies, one needs to specify some defining characteristics of related terms, such as physical activity, exercise, cardiovascular fitness, motor performance and motor learning. *Physical activity* is defined as bodily movements produced by skeletal muscles and incorporates occupational, sports, and other unspecified activities in daily life domains [9]. *Exercise* as a subset of physical activity is characterized as planned, repetitive and done with the purpose to improve physical fitness level [9]. The *physical activity level* is often measured subjectively by standardized physical activity questionnaires that are used to calculate the estimated energy expenditure of a person spent during physical activity and exercise. Less often, it is evaluated objectively by use of activity trackers or pedometers that assess, e.g., number of steps or energy expenditure. The *cardiovascular fitness level* (or: *fitness level*) in turn represents the actual cardiovascular fitness status of a person, often assessed by objective measures to determine participant’s maximal rate of oxygen consumption (VO_2_max). Again, fitness level can be estimated subjectively by self-report. However, objective measures typically have higher accuracy and validity than subjective ones.

The term *motor performance* refers to a temporary status of motor behavior, for example assessed during a motor practice session [68]. In contrast, *motor learning* represents relatively stable changes of the capability to perform a motor task related to practice and aimed interventions [63]. Motor learning – as it is defined in this paper – encompasses the acquisition of new unknown skills or tasks as well as the relearning or improvement of skills acquired in the past. According to Lohse et al. [39], roughly, three time scales of motor learning can be distinguished: short-term (less than one hour of practice), medium-term (one hour to less than 24 h of practice) and long-term practice (more than 24 h to five weeks of practice). Levels of skill acquisition can also be classified with respect to learning phases: the first stage, the *initial phase of learning,* is characterized by fast and high performance improvements within the first practice session. Further stages are an intermediate -, a consolidation -, an automatization - and finally a *retention phase,* in which motor tasks can be performed even after a delay of practice [14, 57].

Further, when examining the association between physical activity and motor performance or learning, we have to distinguish between so-called chronic and acute exercise effects. Studies on chronic exercise investigated long-term exercise effects assessed as an individual’s general *physical activity level, sports participation* or *cardiovascular fitness level* in a cross-sectional or interventional design. In contrast, studies on acute exercise examined the influence of a single bout of exercise (mostly cardiovascular) by use of an experimental (exercisers) and a control group (rest) in an interventional design. When investigating the influence of acute exercise on motor performance, the task performance takes place directly after the exercise. To focus on the influence of acute exercise on motor learning, the task is typically conducted during delayed retention tests (e.g., 24 h later) as motor memory needs time for consolidation ([32] for a review).

### Chronic physical activity and motor performance or learning

A positive association between physical activity and cognitive performance is well established ([26] for a general review; [69] for a meta-analysis with children; [85] for a review regarding respective changes in brain structure and function). Recent approaches seem to attest a positive relationship between chronic engagement in physical activity and motor performance ([28] for a review with children and adolescents), however, research investigating the association between physical activity and motor learning is missing to a large extend ([78] for a review). As several studies have shown that physical activity is associated with enhanced cognitive processing ([11] for a meta-analysis) and even motor tasks require a certain amount of cognitive resources, particularly in older adults [64], motor performance might also be improved by regular engagement in physical activity. The same might be true with regard to motor learning. Particularly the initial phase of learning is characterized by high cognitive loads ([24] for a review) as the phase of automatization is not yet reached [57], and therefore, the initial motor learning processes might also be improved by regular engagement in physical activity.

Recent literature further indicates that regular physical activity or exercise induces biochemical and structural changes that might also benefit motor performance and learning. For example, cardiovascular exercise interventions led to changes in areas of the brain responsible for motor control, indicated by enhanced blood flow in the motor cortex ([88] for young adults), enlarged activity of the sensorimotor network ([84] for older adults),  increased volume of the basal ganglia ([48, 51] for older adults), or enhanced white matter volume ([65] for a review and meta-analysis). Further, coordinative exercise interventions resulted in increased hippocampus volume [50], a brain area known to be involved in motor learning [2, 23] and basal ganglia volume [51]. Changes in hippocampus volume seem to be associated with enhanced levels of brain-derived neurotrophic factor (BDNF) [22, 87]. Dancing led to increased volume of the precentral gyrus – a further area being crucial for motor control – and this enhanced volume was associated with increased levels of BDNF [47].

### Acute exercise and motor performance or learning

The influence of bouts of acute exercise on motor performance (e.g., [79]) and motor learning (e.g., [61, 74]) have been studied extensively in young adults. For example, Roig et al. [61] showed that acute exercise led to improved motor performance 24 h and seven days after initial practice as compared to a resting control condition. Again, underlying mechanisms are still not fully investigated. It is known that exercise induces neurochemical processes like enhanced levels of dopamine, serotonin, norepinephrine, lactate, BDNF, which in turn might facilitate neuroplasticity in the (primary) motor cortex ([70] for a review). Similar neurochemical processes are involved in motor learning [71]. Such neurochemical processes, which take place while the organism adapts to the physical demands of an activity, may already positively impact specific processes directly after an acute bout of exercise. When changes proceed, this might lead to long-term structural changes in the organism.

Up to now, there is no systematic review on the association between acute and chronic physical activity and motor performance or learning in healthy older adults. Thus, we conducted a systematic review to investigate whether an active lifestyle, regular physical exercise or an acute bout of exercise benefit motor performance or learning in upper extremity tasks in healthy older adults. We analyzed whether training effects differed with regard to the study design, type of exercise, and/or the type of motor task.

The main questions to be answered were:

- Is chronic physical activity positively associated with motor performance in upper extremity tasks and does the type of exercise matter?
- Is engagement in chronic physical activity associated with enhanced initial motor learning and/or retention?
- Does a bout of acute exercise facilitate motor performance immediately after exercise?
- Does a bout of acute exercise lead to enhanced initial motor learning and/or retention?

The results of this systematic review have an important impact on understanding the association between physical activity and motor performance or motor learning in older adults. With these results more targeted interventions might be conducted that could be used to maintain motor performance and thus the functionality of older adults.

## Methods

### Database sources and search terms

Based on our research questions, we performed four separate searches, each with a distinct focus of interest on:

(1) Chronic physical activity and motor performance.

(2) Chronic physical activity and motor learning.

(3) Acute exercise and motor performance.

(4) Acute exercise and motor learning.

The search strategy was reviewed by experts in the field of physical activity, motor performance and motor learning. The four electronic databases Cochrane library, PubMED, PsycINFO and Scopus were searched systematically. The last search update was conducted at December 9th, 2016. Each database was searched individually, but all searches were done with a consistent search strategy and search terms (cf., Tables 1, 2, 3 and 4). The search was limited to English language peer-reviewed articles and the publication years from 1990 to present.

**Table 1:** Search terms: chronic physical activity and motor performance

Level	Category	Search terms
1	Physical Activity	“physical activity” or “fitness” or “physical fitness” or “physical exercise” or “exercise” or “energy expenditure” or “sport” or “endurance”
	+ connected	AND
2	Motor performance	“motor performance” or “motor task” or “motor skill” or “fine motor control” or “fine motor performance” or “dexterity” or “manual dexterity” or “force control” or “visuomotor* tracking” or “motor control” or “movement control” or “manual performance” or “grip force” or “finger movement” or “voluntary movement”
	+ connected	AND
3	Older adults	“old* age” or “advanced age” or “old* adults” or “elderly” or “senior*” or “aging” or “ageing”

**Table 2:** Search terms: chronic physical activity and motor learning

Level	Category	Search terms
1	Physical Activity	“physical activity” or “fitness” or “physical fitness” or “physical exercise” or “exercise” or “energy expenditure” or “sport” or “endurance”
	+ connected	AND
2	Motor learning	“motor learning” or “motor adaptation” or “motor skill learning” or “skill learning” or “skill training” or “motor training” or “motor skill acquisition” or “skill acquisition” or “motor improvement” or “short-term learning” or “motor sequence learning” or “motor memory” or “motor consolidation”
	+ connected	AND
3	Older adults	“old* age” or “advanced age” or “old* adults” or “elderly” or “senior*” or “aging” or “ageing”

**Table 3:** Search terms: acute exercise and motor performance

Level	Category	Search terms
1	Acute exercise	“acute exercise” or “acute* exercise” or “fatigue” or “physical stress” or “intermittent* exercise” or “after exercise” or “acute stress”
	+ connected	AND
2	Motor performance	“motor performance” or “motor task” or “motor skill” or “fine motor control” or “fine motor performance” or “dexterity” or “manual dexterity” or “force control” or “visuomotor*tracking” or “motor control” or “movement control” or “manual performance” or “grip force” or “finger movement” or “voluntary movement”
	+ connected	AND
3	Older adults	“old* age” or “advanced age” or “old* adults” or “elderly” or “senior*” or “aging” or “ageing”

**Table 4:** Search terms: acute exercise and motor learning

Level	Category	Search terms
1	Acute exercise	“acute exercise” or “acute* exercise” or “fatigue” or “physical stress” or “intermittent* exercise” or “after exercise” or “acute stress”
	+ connected	AND
2	Motor learning	“motor learning” or “motor adaptation” or “motor skill learning” or “skill learning” or “skill training” or “motor training” or “motor skill acquisition” or “skill acquisition” or “motor improvement” or “short-term learning” or “motor sequence learning” or “motor memory” or “motor consolidation”
	+ connected	AND
3	Older adults	“old* age” or “advanced age” or “old* adults” or “elderly” or “senior*” or “aging” or “ageing”

### Study selection and eligibility criteria

We included studies on healthy older adults (≥ 60 years of age), with no brain injuries, no cognitive declines or intellectual disabilities, and no chronic diseases (e.g., Parkinson’s disease, motor impairment, stroke). Further, we included studies investigating motor performance or learning by use of tasks (1) performed with upper extremities (i.e., finger, hand or arm) and (2) with a complex motor component, referring to the portion of involved subsystems or abilities (cf. [83]; i.e., no simple or choice reaction time tasks, no muscular strength tests) and/or (3) containing a motor speed component. In this understanding speed tapping requires fast motor organization and expresses the maximum frequency of impulses, which can be send by motor areas [72]. Simple and/or discrete key pressing tasks have not be considered as they require a very simple/less-complex motor action.

**Fig. 1:**
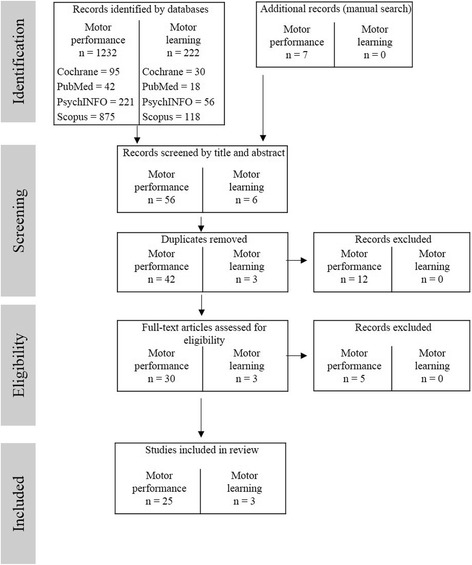
Flow chart through the different phases of the review for chronic physical activity and motor performance as well as chronic physical activity and motor learning

We included longitudinal studies, randomized controlled trails (RCT’s), controlled pre-post designs (without a randomization procedure) and cross-sectional studies as well as relevant fitness group and/or age comparison studies. The authors screened the title and the abstract of the selected articles for the inclusion criteria. For chronic exercise, a total of 1454 studies was identified from the database search. The flow of studies through the review is summarized in Fig. 1. Based on the abstracts (n = 45 after screening and duplicate removement), 12 studies were excluded as they used simple or choice reaction time tasks (*n* = 3; [4, 30, 59]), focused on lower extremities (*n* = 5; [25, 38, 53, 60, 90]), compromised middle-aged participants only (*n* = 2; [43, 44]), described no behavioral but only brain data (*n* = 1; [48]) or described the methodological process of the study only (*n* = 1; [59]). Full articles were retrieved if they were relevant, or if it was unclear, whether they were relevant after reading the abstract. We assessed 30 articles focusing on motor performance and three articles focusing on motor learning for eligibility (total *n* = 30 as three papers: [19*, 21*, 89*] were used for motor performance and motor learning). Two articles were excluded for research on the association between chronic exercise and motor performance, because they reported results with respect to motor learning only [21*, 89*], one paper was excluded as it focused on lower extremities [31], one because it reported only a global measure of motor function including lower extremity tasks [8] and one because it incorporated only physically active older adults and no control group [10]. Finally, a total of 27 studies, ranging from the year of publication 1994 to 2016, met all inclusion criteria and were included in the review. 25 of these studies examined research questions with respect to the association between chronic physical activity and motor performance and three investigated the association with motor learning (one paper: [19*] was used for both).

**Fig. 2:**
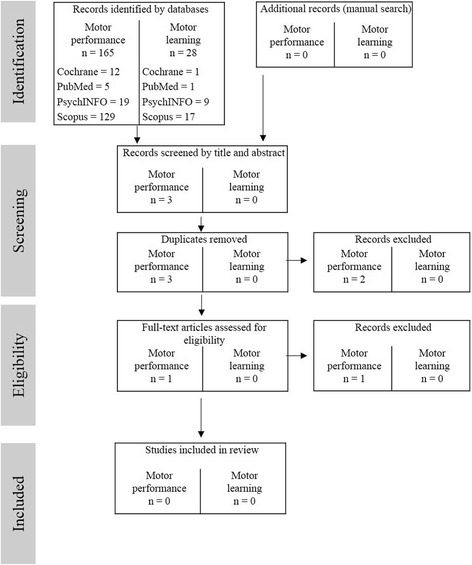
Flow chart through the different phases of the review for acute exercise and motor performance as well as acute exercise and motor learning

For acute exercise, a total of 193 studies was identified from the database search (Fig. 2). No study was found meeting the inclusion criteria with respect to the influence of a bout of acute exercise on motor performance or motor learning in older adults (Fig. 2).

## Results

### Chronic physical activity and motor performance

As characteristics and results of studies about chronic physical activity and motor performance differed, results of the systematic review were summarized with respect to sample characteristics, study design, types of exercise and motor tasks.

#### Sample characteristics

Main characteristics of the study samples are summarized in Table 5. The total number of participants was 1399, at least 679 were females (about 49%; four studies made no specification about gender). 1076 participants were older adults (at least 54% women) and 256 were in young comparison groups (at least 24% women). The age range of included older adults was 60 to 94 years of age, the age range of young adults was 18 to 30 years of age. With respect to age, generally, young adults performed better in the respective motor task. Only one study investigated gender effects [56*] and found no significant differences in a five-month exercise intervention.

**Table 5:** Overview of studies on chronic physical activity and motor performance

Author, Year	Participants	Motor task	Other depended variables	Method to assess physical activity	Design and statistics	Results
[1*]	OA: *n* = 30, f = 14, n/a, 76.4 ± 5; YA: *n* = 12, f = 6, n/a, 22.1 ± 2	Manual dexterity (Purdue pegboard), hand-arm movements (wrist-position matching), L + R	Grip strength	Sj: PAQ (IPAQ)	Cross-sectional, median split: energy expenditure, active OA (≥ 2900 kcal/wk) vs. inactive OA (< 2800 kcal/wk) vs. YA	Physical active OA > sedentary OA in hand-arm movements. No effect of PA-levelon manual dexterity.
[3*]	All OA: IG: *n* = 5, f = 3, 72–91, 80.2 ± 7.8; CG: *n* = 5, f = 3, 83–89, 84.8 ± 2.5	Force matching task (force tracking with index finger), D	/	Oj: CFT (submaximal graded exercise tolerance)	Interventional, IG (8 wk. low-intensity AE) vs. CG (n/a)	IG ↑ in force matching task, besides an aerobic training effect.
[7*]	All OA: active: *n* = 20, f = 10, 67–85, n/a; inactive: *n* = 20, f = 12, 67–85, n/a	Manual dexterity (Minnesota Manual Dexterity Test), n/a	Nelson Hand Reaction Test	N/a	Cross-sectional, active OA vs. inactive OA	Active OA > inactive OA in manual dexterity.
[12*]	OA tennis: *n* = 21, f = 10, 60–82, 67.3 ± 5.3; OA runners: *n* = 23, f = 10, 61–77, 68.0 ± 5.2; OA exerciser: *n* = 20, f = 10, 63–79, 68.2 ± 5.1; YA: *n* = 20, f = 10, 19–29, 21.8 ± 2.4	Manual dexterity (thumb & finger sequence), tapping speed (tapping task with a stylus), hand-arm movements (matching a ball position with wrist), D	Balance, CRT, SRT	Sj: PAQ (modified Baecke), Oj: CFT (Rockport Fitness Walking Test, estimated VO_2_max)	Cross-sectional, type of activity in OA (tennis vs. runners vs. exerciser) vs. YA	No sig. difference between the different kinds of sports. No sig. correlations between estimated VO_2_max and motor performance (regardless of kind of sport).
[19*]	OA: *n* = 41, f = 0, 60–80, n/a; YA: *n* = 43, f = 0, 20–30, n/a	Hand-arm movements (mirror tracing task), R	EEG (alpha activity)	Sj: PAQ (modified Baecke), Oj: CFT (submaximal bicycle test, estimated VO_2_max)	Cross-sectional, median split: estimated VO_2_max, YA median: 41.11 ml/kg/min, OA median: 26.01 ml/kg/min	No effect of cardiovascular fitness on hand-arm movements.
[29*]	All OA: swimmer: *n* = 20, f = 12, n/a, 65.4 ± 5.5; active CG: *n* = 34, f = 21, n/a, 67.4 ± 5.7	Hand-arm movements (sequential pointing task), D	Static postural stability test	Sj: Exercise screening questionnaire	Cross-sectional, swimming (≥ 500 per session, 3 session/wk., ≥ 3 years) vs. active CG (jogging or mountain climbing ≥3 times/wk)	Swimming group > active control group in hand-arm movements.
[33*]	All OA: IG: *n* = 25,f = 17, 60–94, 68.60 ± 1.45; CG: *n* = 10, f = 7, 60–94, 72.30 ± 1.84	Hand-arm fine motor battery: maintain arm-hand position (steadiness), aiming, motor dexterity(pin plugging), wrist-finger movements (tapping), L + R	Cognitive performance, posture, RT tasks, tactile performance	Sj: PAQ (ECQ), Oj: CFT (spiroergometry, VO_2_peak)	Interventional, IG (24 wks, 1 h/wk. dance program) vs. inactive CG	IG ↑ in steadiness (L), aiming (time, L+ R), pin plugging (L + R), tapping (R). No effect of dance intervention on steadiness (R), aiming (error R; tendency for error, L: *p* = .051), tapping (L). CG ↑ in tapping (L). Total motor performance score: IG ↑, tendency CG ↑ (*p* = .073).
[34*]	All OA: expert dancer: *n* = 11, f = 5, 60–94, 71*.*18 ± 1*.*13; nondancer, inactive CG: *n* = 38, f = 30, 60–94, 71*.*66 ± 1*.*11	Hand-arm fine motor battery (see [33])	Balance & gait control, cognitive performance, posture, RT tasks, tactile performance	Sj: PAQ (ECQ)	Cross-sectional, expert dancer group vs. nondancer, inactive CG	Expert dancer > CG in aiming (error, R; tendency for time, R: *p* = .059), pin plugging (R). No effect of expert dancing on steadiness (L + R), aiming (error + time, L), pin plugging (L), tapping (R, tendency for L: *p* = .057). Total motor performance score: tendency expert dancer > CG (*p* = .080).
[35*]	All OA: amateur dancer *n* = 24, f = 19, 65–84, 71.69 ± 1.15; inactive CG: *n* = 38, f = 30, 61–94, 71.66 ± 1.13	Hand-arm fine motor battery (see [33])	See [34*]	Sj: PAQ (ECQ)	Cross-sectional, amateur dancers (16.4 ± 12.7 years of experience, 1.33 ± 0.24 h/wk) vs. inactive CG (without dancing or sport activities)	Amateur dancer > CG in steadiness (L), aiming (error, R), tapping (L). No effect of amateur dancing on steadiness (R), aiming (error + time, L; time, R), pin plugging (L + R), tapping (R). Total motor performance score: amateur dancer > CG.
[36*]	All OA: IG: *n* = 7, f = 0, 70–80, 75 ± 2; CG: *n* = 4, f = 0, 70–80, 76 ± 2	Force matching task (finger pinch force control: constant + sinus; 20% & 40% of MVC), L + R	Upper limb strength	Not assessed	Interventional, IG (strength training: 6 wks, 2 d/wk., 4 sets of 3 exercises: biceps curls, wrist extension) vs. CG (no training)	IG > CG at high constant force and sinusoidal force production in the trained limb. No effect of strength training on low constant force. No effect for the untrained limb.
[37*]	Sportive OA: *n* = 27, f = 0, n/a, 63.75 ± 5.02; nonsportive OA: *n* = 24, f = 0, n/a, 61.88 ± 4.67; sportive YA: *n* = 24, f = 0, n/a, 20.71 ± 3.18; nonsportive YA: *n* = 23, f = 0, n/a 22.13 ± 2.05; martial arts OA: *n* = 22, f = 0, n/a, 62 ± 5.89; martial arts YA: *n* = 23, f = 0, n/a 22.22 ± 2.88	Force matching task (force tracking task, index finger; constant: 5/25% MVC + sinus: 5–25% MCV), tapping speed (tapping task, index finger), L + R	Cognitive performance, posture	Sj: Self-reported regular exercise per week	Cross-sectional, active (regular weekly activities; YA/OA) vs. inactive (YA/OA) vs. martial arts YA/OA)	Inactive > martial arts in all force tracking conditions, no difference in sinus task. No influence of activity group on tapping speed.
[52*]	All OA: 1: *n* = 6, f = 3, n/a, 76 ± 6; 2: *n* = 6, f = 3, n/a, 76 ± 6	Manual dexterity (Grooved Pegboard Test & Jebsen Taylor Hand Function Test), force matching task (four fingers, ramp from resting level to 25% of MVC), L + R	Maximum force production task	Not assessed	Interventional, strength training: maximal finger pressing force, 6 wks, 2 times/d, 2 × 10 repetitions of 2 s), different training groups: (1)“right-distal site + left-proximal site training vs. (2) “left-distal site + right- proximal site”	Both training groups ↑ in Grooved Pegboard test, but not in Jebsen Taylor Hand function test. Tendency for ↑ in force tracking task.
[54*]	All OA: Tai chi: *n* = 22, f = 14; n/a, 67.8 ± 5.1; CG: *n* = 20, f = 15, n/a, 68.1 ± 5.2	Hand-arm movements (sequential pointing task), n/a	/	Sj: Self-reported engagement in PA	Cross-sectional, tai chi group (1–2 h, 5–7 d, > 3 y) vs. active CG (≥ 3 exercise/wk)	Tai chi group > CG (in four out of five parameter) in hand-arm movements.
[56*]	All OA 60–82, 67 ± n/a: gymnastics: *n* = 19, f = 15, n/a, n/a,; swimming: *n* = 11, f = 9; n/a, n/a; senior dance: *n* = 15, f = 12, n/a, n/a; CG: *n* = 14, f = 6	Hand-arm movements (hand and arm coordination in the clothes-pin test), L + R	Balance, blood samples, dietary habits, flexibility, SRT	Oj: CFT (exercise test on treadmill, estimated VO_2_max)	Interventional, 5 months: IG 1: multi-component (45 min, 2 times/w: aerobic, strength, balance & flexibility, 65% mHR) vs. IG 2: swimmer (25 min, 2 times/w: water gymnastic + swimming. 65% mHR) vs. IG 3: senior dance (45 min, 2 times/w, 58% of mHR) vs. CG (no training), each separated in female & male	All exercise groups ↑ in hand-arm movements, no difference between female and male. Male CG ↑ with dominant hand.
[66*]	All OA: IG: *n* = 61, f = n/a, 60–88, 73.7 ± 6.5; CG: *n* = 49, f = n/a, 62–91, 77.9 ± 7.8	Manual dexterity (Pegs-over test), L + R	Balance & gait, grip strength, walking	Not assessed	Interventional, IG (low intensity multi-component exercise: stretching, flexibility, range of motion exercises, 30 min, 2 times/week, 1 year)	No ↑ of IG in manual dexterity.
[67*]	Fit OA: *n* = 19, f = n/a, 60–73, 63.7 ± 3.8; non fit OA: *n* = 19, f = n/a, 60–73, 66.4 ± 4.2; fit MA: *n* = 17, f = n/a, 35–45, 40.2 ± 3.7; non fit MA: *n* = 18, f = n/a, 35–45, 40.0 ± 3.0; fit YA: *n* = 15, f = n/a, 18–25, 21.8 ± 3.3; non fit YA: *n* = 17, f = n/a, 18–25, 23.5 ± 3.3	Tapping speed (finger tapping task), R	Battery of cognitive tasks	Oj: CFT (submaximal bicycle ergometer test, predicted VO_2_max)	Cross-sectional, median split: VO_2_max. YA: 44.9 ml/kg/min, MA: 33.1 ml/kg/min, OA: 25.4 ml/kg/min	No effect of fitness on tapping speed.
[73*]	Active OA1: *n* = 10, f = 10, 70–79, n/a; inactive OA1: *n* = 9, f = 9, 70–79, n/a; active OA2: *n* = 16, f = 16, 60–69, n/a; inactive OA2: *n* = 14, f = 14, 60–69, n/a; active MA: *n* = 18, f = 18, 50–59, n/a; inactive MA: *n* = 14, f = 14, 50–59, n/a; active YA: *n* = 10, f = 10, 20–29, n/a; inactive YA: *n* = 20, f = 20, 20–29, n/a	Tapping speed (stationary tapping; between target tapping), D	Digit Symbol Substitution, DRT, SRT, Trailmaking test	Sj: Self-reported amount of activity	Cross-sectional, active (walked/jogged/ran ≥3 miles/d, 3 d/wk., ≥ 5 years) vs. inactive (n/a) and age groups (OA1: 70–79, OA2: 60–69, MA: 50–59, YA: 20–29)	No effect of cardiovascular exercise on tapping speed.
[80*]	Active OA: *n* = 12, f = 7, n/a, 65.1 ± 0.9; inactive OA: *n* = 12, f = 8, n/a, 65.3 ± 1.1; active YA: *n* = 11, f = 5, n/a, 22.9 ± 0.5; inactive YA: *n* = 11, f = 5, n/a, 22.0 ± 0.6	Hand-arm movements (flexion and extension aiming wrist movements), R	/	Sj: PAQ (Baecke)	Cross-sectional, median split: Baecke score, active OA = 9.3 ± 0.2, inactive OA = 6.9 ± 0.3; active YA = 9.7 ± 0.3, inactive YA = 6.7 ± 0.3	No difference betweenphysically active OA and inactive OA in hand-arm movements.
[81*]	Fit OA: *n* = 13, f = 8, 61.5–65.5, 64.9 ± n/a; non fit OA: *n* = 13, f = 6, 61.4–68.2, 65.7 ± n/a; YA: *n* = 15, f = 9, 23.3–23.6, 23.5 ± n/a	See [80*]	/	Sj: PAQ (Baecke)	Cross-sectional, median split: Baecke score, active OA = 7.8 (7.6–8.8), inactive OA = 6.1 (5.3–6.9), YA = 7.9 (7.1–8.5)	Physically active OA > inactive OA in hand-arm movements.
[82*]	OA tai chi practitioners: *n* = 12, f = n/a, n/a, 67.75 ± 7.57; OA non-practitioners: *n* = 11, f = n/a, n/a, 65.85 ± 6.34; YA: *n* = 12, f = n/a, n/a, 23.58 ± 4.19	Hand-arm movements (cued, flexion- and abduction-reaching task), n/a	Stand-reaching task	Sj: PAQ (PASE)	Cross-sectional, long-term practice of Tai Chi (≥ 100 h practice in the last y) vs. non-practitioners	Tai chi group > non- practitioners in hand-arm movements.
[86*]	All OA: high active: *n* = 19, f = 19, 60–80, 70.8 ± 4.4; moderately active: *n* = 15, f = 15, 60–80, 71.9 ± 3.9; inactive: *n* = 16, f = 16 72.1 ± 4.3	Manual dexterity (Minnesota test), L + R	Balance, blood pressure, flexibility (hip, spine, shoulder), grip strength, peak expiratory flow, RT, walking	Sj: PAQ (modified Baecke)	Cross-sectional, high active vs. moderately active vs. inactive (separated in 3 tertials)	No influence of high PA level on manual dexterity.
[92*]	All OA: tai chi: *n* = 20, f = 8, n/a, 65.4 ± 5.5; swimmer: *n* = 32, f = 20, n/a, 67.0 ± 6.6; active CG: *n* = 34, f = 21, n/a, 67.4 ± 5.7	Hand-arm movements (sequential pointing task), D	Balance, postural stability	Sj: Self-reported engagement in PA	Cross-sectional, tai chi group (approximately 54 min/training, ≥ 3 times/wk) vs. swimming group (≥ 500 per session, 3 session/wk., ≥ 3 years) vs. active CG (non-swimmers, no tai chi)	Tai chi & swimming group > CG in hand-arm movements, tai chi & swimming group: no difference.
[93*]	All OA *n* = 38, f = 29: tai chi: *n* = 28, n/a, 76–89, 78.8 ± 2.1; locomotor activity: *n* = 10, n/a, 76–89, 79.2 ± 1.9	Hand-arm movements (aiming task), R	Balance	Not assessed	Interventional, exercise program (8 wks, 3 times/wk., ≥ 45 min): tai chi vs. locomotor activity (walking or jogging)	Tai chi: bigger ↑ in arm movement smoothness. Both groups: no ↑ in arm movement speed.
[94*]	All OA: tai chi: *n* = 12, f = 9, 76–88, 79.3 ± 2.4; locomotor activity: *n* = 8, f = 6, 76–88, 79.5 ± 1.9	See [93*]	/	Sj: Self-reported engagement in PA	Interventional, see [93]	Tai chi group more ↑ than CG in hand-arm movements.
[95*]	All OA: IG: *n* = 15, f = 10, 60–83, 69.43 ± 6.17; CG: *n* = 15, f = n/a, n/a, n/a: matched to IG	Manual dexterity (Motor Performance Series of the Vienna System Series), D	Balance, flexibility, grip strength, whole-body reaction time	Not assessed	Interventional, IG (low-moderate aerobic exercise, 60 min, 3 times/ week, 9 months) vs. CG (n/a)	IG ↑ in manual dexterity, CG no difference (tendency for performance decline).

Legend: *AE* aerobic exercise, *CFT* cardiovascular fitness test, *CRT* choice reaction time, *d* day(s), *D* dominant hand, *DRT* discrimination reaction time, *ECQ* everyday competence questionnaire, *EEG* electroencephalography, *f*= female, *h* hour(s), *IG* intervention group, *IPQ* international physical activity questionnaire, *L* left, *OA* older adults, *MA* middle-aged, *mHR* maximum heart rate, *n/a* not specified, *Oj* objective measure, *PAQ* physical activity questionnaire, *PASE* physical activity scale for the elderly, *R* right, *sig*. significant, *RT* reaction time, *Sj* subjective measure, *SRT* simple reaction time, *y* year, *YA* young adults, *wk.* week, *EG* > *CG* EG better CG, *EG* < *CG* EG worse than CG, ↑ = improvement

#### Study design

16 of the 25 identified studies investigated the effects of exercise on motor performance in older adults by use of a *cross-sectional design*. Therefore, studies examined either different subgroups of older adults, i.e., active/fit vs. inactive/unfit adults (*n* = 9: [1*, 7*, 19*, 37*, 67*, 73*, 80*, 81*, 86*]), different cardiovascular activities (*n* = 1: [29*]) and/or different kinds of motor-coordinative/sports activities (*n* = 7: [12*, 34*, 35*, 37*, 54*, 82*, 92*]; see section “type of exercise”). Nine of the cross-sectional studies also compared the sample of older adults to young participants [1*, 12*, 19*, 37*, 67*, 73*, 80*–82*]. The remaining nine of 25 studies used an *interventional design* with an experimental and control group. No intervention study encompassed a group of young participants. Motor performance was assessed at baseline and after a targeted intervention of different kinds of physical/sports-activities (*n* = 9: [3*, 33*, 36*, 52*, 56*, 66*, 93*–95*]; see section “type of exercise”). The interventions compromised durations from six weeks to one year. The lengths of the intervention did not seem to effect results systematically, but intervention studies seem to be more promising than cross-sectional studies (cf. below).

#### Type of exercise

##### Physical activity level and/or cardiovascular fitness as independent variable

Different methods were used to assess physical activity level and/or cardiovascular fitness in 19 studies. In this section we refer to the ten (out of 16) cross-sectional studies that used these measures as independent variables to determine effects on motor performance: Seven studies assessed the physical activity level of the participants subjectively, four by use of physical activity questionnaires, i.e., by the (modified) Baecke habitual physical activity questionnaire (*n* = 3; [80*, 81*, 86*]) or the International Physical Activity Questionnaire (IPAQ, *n* = 1; [1*]) and three studies did not specify which tool they used to assess engagement in physical activity [7*, 37*, 73*]. Only in two of these seven studies using subjective measures, active older adults revealed better motor performance than inactive older adults [7*, 81*], whereas in four studies such an effect was not present [37*, 73*, 80*, 86*], and one study revealed ambiguous results ([1*]; positive effects for hand-arm movements and no effect for manual dexterity). Only three cross-sectional studies calculated the association between cardiovascular fitness and motor performance by use of objective fitness tests. This was done by spiroergometry [19*, 67*], or the Rockport Fitness Walking Test [12*]. All three studies reported no difference between high and low fit older adults [19*, 67*], or no correlation between estimated VO_2_max and motor performance ([12*]; they additionally investigated the effect of tennis, cf., below).

#### Targeted exercise as independent variable

In addition to or instead of assessing the physical activity or cardiovascular fitness level, seven of the cross-sectional studies examined whether effects of exercise differed with regard to the type of exercise (cf., study design). Also all nine intervention studies investigated effects of different types of physical exercise or specific intervention programs [3*, 33*, 36*, 52*, 56*, 66*, 93*–95*]. In the following we summarized results with respect to type of exercise.

Three studies investigated the effect of *cardiovascular exercise* (combination of calisthenics, walking/dancing steps, and cycling, or swimming) on motor performance [3*, 29*, 95*]. Twelve studies used kinds of *motor-coordinative exercises* like a multi-component training [12*, 56*, 66*], dancing [33*–35*] or martial arts including tai chi [37*, 54*, 82*, 92*–94*]. Two more studies used upper-limb specific *strength training* [36*, 52*].

##### Cardiovascular exercise

Contrary to the majority of cross-sectional studies that assessed cardiovascular fitness (cf., above), both studies that conducted a cardiovascular exercise intervention [3*, 95*] found beneficial effects of cardiovascular exercise. This is notable as study design, intervention length and intensity as well as motor outcomes considerably differed between studies. Interestingly, one of the cardiovascular exercise studies additionally assessed the cardiovascular fitness level and found no significant change in the fitness level [3*], therewith somehow confirming the reported cross-sectional results ([56*] for supporting results). Hsu et al. [29*] compared two types of cardiovascular exercises and revealed that swimmer performed better in a hand-arm movement task than persons who were regularly engaged in jogging/mountain climbing (referred to as an active control group).

##### Motor-coordinative exercises

Sharpe et al. [66*] used a one year low intensity *multi-component training* containing mobility and flexibility exercises executed while sitting and standing and found no improvement in a bilateral fine motor coordination task. In contrast to Sharpe et al. [66*], Puggaard and colleagues [56*] claimed an improvement after five months intervention. Furthermore, Puggaard et al. [56*] compared the multi-component training to cardiovascular exercises and dancing, but did not find differences between different types of training [12*, 56*]. Interestingly, all groups improved without a change in cardiovascular fitness (estimated VO_2_max). In the same vein the cross-sectional study by Dascal and Teixeira [12*] found no difference between long-term participation in multi-component exercises (i.e., aerobic fitness, strength training and flexibility), cardiovascular exercise (i.e., running) and tennis.

Kattenstroth and colleagues published a series of papers regarding the association between *dancing* and motor performance in older adults [33*–35*]. They compared dancers (amateur dancer: [35*]; expert dancer: [34*]) with an inactive control group without dancing experience in a hand-arm fine motor battery. In a third study, they conducted a 24-week dancing program ([33*], no change of cardiovascular fitness (VO_2_peak)). In all three studies, the motor performance score of the whole motor battery pointed to a positive effect (significant or marginally significant) of dancing [33*–35*]. However, the results of the particular subtasks did not reveal a consistent pattern (see Table 5).

Four cross-sectional studies [37*, 54*, 82*, 92*] and two interventional studies [93*, 94*], investigated *martial arts*, five of them focused on tai chi (all except [37*]). Studies focusing on tai chi revealed an advantage of tai chi training as compared to an active control group [54*, 92*] or non tai chi practitioners [82*]. When comparing tai chi to different cardiovascular activities, results differed. No difference was found between a tai chi and swimming group [92*], but between tai chi and cardiovascular exercise [93*, 94*]. That is, an eight-week tai chi intervention revealed positive effects on movement speed, smoothness and movement variability in different manual aiming tasks, whereas cardiovascular exercise (locomotor activities, i.e., walking or jogging) did not [93*, 94*]. Total arm movement speed was not altered in any of the groups [93*]. Only one motor-coordinative exercise study stated non-significant effects: Krampe et al. [37*] found no association between martial arts experience as well as general physical activity behavior of older adults and visuospatial accuracy or with psychomotor speed. The authors even reported that inactive older adults revealed better motor performance in visuospatial accuracy [37*].

##### Strength training

Two intervention studies focused on strength training of arm, wrist, and/or finger and revealed a positive association between strength exercise and upper extremity performance. Keogh et al. [36*] found positive effects of strength training on finger pinch force control at high constant and sinusoidal force production in the trained limb [36*], but no effect on low constant force or in the untrained limb [36*]. Olafsdottir et al. [52*] reported a significant improvement in the Grooved Pegboard Test and a positive tendency in a force tracking task, but not in the Jebsen Taylor Hand Function Test [52*].

#### Type of motor task

Although we focused on upper extremity motor performance tasks only, tasks differ with respect to their demands during execution. Upper extremity motor tasks included in studies of this review can be categorized into four groups: (1) hand-arm movements, (2) force matching tasks, (3) manual dexterity tasks and (4) speed tapping tasks.

1. In *hand-arm movement tasks* (*n* = 13) participants have to move their hand, wrist or arm to a certain target (i.e., aiming tasks). Regardless of the independent variable (type of exercise), the majority of studies (*n* = 7) reported a positive association between hand-arm movement performance and chronic physical activity level [1*, 12*, 81*], tai chi [82*, 92*], swimming [29*, 82*], or after a five months intervention (all three intervention groups, [56*]). However, two studies reported no differences between active and inactive [80*] or fit and unfit older adults [19*]. Van Halewyck et al. [80*] did not find a positive association with the exact same wrist aiming task as in the Van Halewyck et al. [81*] paper. However, the active older adults of the Van Halewyck et al. [81*] study (Baecke score of 9.3 ± 0.2) had a higher level of physical activity than the active older adults of the Van Halewyck et al. [80*] study (Baecke score mean 7.8; range 7.6–8.8), which might point to an influence of the amount of physical activity. Four studies revealed ambiguous results, i.e., results differed regarding whether the left or right hand performed the task [33*–35*] and/or with respect to the analyzed parameter [33*–35*, 54*].
2. *Force matching tasks* (*n* = 4) require participants to adjust their finger forces to a given target force so that fine adjustment of the corresponding muscles is required (i.e., precision tasks). Results with respect to force matching tasks are very dissimilar. A cardiovascular exercise program induced better precision task performance [3*], but a cross-sectional study did not find differences between active and inactive older adults [37*]. Strength training improved force control at high constant force and sinusoidal force production [36*], but not at low constant force [36*] and not in a ramp force profile [52*].
3. Results with respect to *manual dexterity* (*n* = 8) are also mixed: One publication described a positive association between the physical activity or fitness level and manual dexterity by use of the Minnesota Manual Dexterity Test [7*]. Two intervention studies reported improved manual dexterity after a finger-strength training (Grooved Pegboard test [52*]), a cardiovascular exercise program (Motor Performance Series of the Vienna System Series [95*]) or dancing intervention (pin plugging [33*]). However, in five studies no differences in performance between active and inactive older adults (Purdue pegboard test [1*]; finger dexterity task [12*]; the Minnesota test [86*]) or dancers and non-dancers (pin plugging [34*] (left hand); [35*]) were found and no effect through strength (Jebsen Taylor Hand function test [52*]) or multi-component training (the Pegs-over test [66*]) was examined in two further studies.
4. Studies in the third category of *speed tapping tasks* (*n* = 7) were all assessed by (finger) tapping tasks, which require participants to tap a stylus as fast as possible in one or two predefined target spaces for a given time (10 s to 32 s in this review). No study found differences between active and inactive [37*, 73*], or fit and unfit older adults [67*]. The type of exercise did not influence results as shown for a comparison between tennis players, runners and general exercisers [12*]. Results with respect to dancing were mixed [33*–35*].

Taken together, the most consistent positive results have been shown for hand-arm movements (but still ambiguous), whereas the less promising results were revealed for speed tapping and manual dexterity tasks. Results for force matching tasks were very inconsistent.

### Chronic physical activity and motor learning

#### Sample characteristics

Characteristics of the sample are summarized in Table 6. 150 participants took part in all three motor learning studies (at least 18 were females: one study made no specification about gender [89*], one study included only men [19*]. Eighty-seven participants were older adults (at least 9% women) and 63 young adults (at least 16% women). The age range of included older adults was 60 to 80 years of age, the age range of young adults was 20 to 40 years of age.

**Table 6:** Overview of studies on chronic physical activity and motor learning

Author/ Year	Participants	Motor task	Other depended variables	Method to assess physical activity	Design and statistics	Results
[19*]	OA: *n* = 41, f = 0, 60–80, n/a.; YA: *n* = 43, f = 0, 20–30, n/a	Visuospatial accuracy (Mirror tracing task, R)	EEG (alpha activity)	Sj: PAQ (modified Baecke), Oj: CFT (sub-maximal bicycle test, estimated VO_2_max)	Acquisition (175 trials), retention (2 d later, 20 trials). Fitness: median split: estimated VO_2_max, YA median: 41.11 ml/kg/min, OA median: 26.01 ml/kg/min	Positive association of cardiovascular fitness and acquisition of mirror tracing. No association of cardiovascular fitness and retention.
[21*]	OA: *n* = 18, f = 8, 60–80, 68.0 ± 5.9; YA: *n* = 20, f = 10, 20–40, 24.2 ± 5.2	Visuospatial accuracy (Mirror tracing task, R)	EEG (alpha activity)	Oj: CFT (submaximal bicycle test, estimated VO_2_max)	Acquisition (approximately 87 trials), retention (24–72 h later, 40 trials. Fitness: regression analyses of VO_2_max and motor learning	Positive association of cardiovascular fitness and acquisition and retention of a mirror tracing.
[89*]	All OA: fit: *n* = 14, 64–76, n/a; non fit: *n* = 14, 64–76, n/a.	Rapid arm reaching movements (motor adaptation/ visuomotor rotation)	/	Sj: PAQ (Stanford Brief Activity Survey), Oj: accelerometer	Baseline (80 trials), training (192 trials), transfer (192 trials). Physical activity: active (≥ 30 min, ≥ 3 d/wk. of AE) vs. inactive (≤ 2 d/wk. low-intensity exercise)	Active OA showed similar motor adaptation pattern to YA (asymmetrical transfer), inactive OA revealed a different pattern (symmetrical transfer).

Legend: *CFT* cardiovascular fitness test, *d* day(s), *EEG* electroencephalography, *f* = female, *h* hour(s), *L* left, *OA* older adults, *Oj* objective measure, *n/a* not specified, *PAQ* physical activity questionnaire, *R* right, *sig*. significant, *Sj* subjective measure, *y* year, *YA* young adults, *wk.* week

#### Study design

Only three studies investigating the association between chronic physical exercise and motor learning in upper extremity motor tasks were identified by the literature search [19*, 21*, 89*]. All studies used a pre-to-post-test design comparing different groups of older adults and assessed physical activity/cardiovascular fitness cross-sectionally. The studies by Etnier and Landers [19*] and Etnier et al. [21*] also contained a group of young participants. Motor practice times of all studies belong to the time scale of short-term motor learning [19*, 21*, 89*].

#### Type of exercise

Two studies assessed cardiovascular fitness objectively and one cardiovascular exercises behavior on behalf of a physical activity questionnaire. Etnier & Landers [19*] classified cardiovascular fitness by a median split of the estimated VO_2_max, assessed by a submaximal bicycle test. Etnier et al. [21*] conducted a regression analysis of the VO_2_max and motor learning output and Wang et al. [89*] regarded participants as active and inactive with respect to their self-reported engagement in physical activity (active: at least 30 min on three days per week of cardiovascular exercise; inactive: less than two days per week of low-intensity exercise).

#### Type of motor task and stage of motor learning

All motor learning studies used tasks that can be classified into the group of hand-arm movements. All studies found a positive effect of cardiovascular fitness or physical activity level on initial phase of motor learning in a mirror tracing task ([19*]: acquisition: 175 trials, approximately 48 min; [21*]: acquisition: mean about 87 trials, approximately 12 min) and motor adaptation in a visuomotor rotation task requiring rapid arm reaching movements ([89*]: baseline: 80 trials each hand, training: 192 trials, transfer: 192 trials). The first study by the research group of Jennifer Etnier found no effect on retention ([19*]: two days later, 20 trials, approximately 3 min), whereas the second study revealed that higher cardiovascular fitness was associated with enhanced retention ([21*]: 24–72 h later, 40 trials, approximately 5.5 min). Wang et al. [89*]: did not assess retention explicitly.

Taken together, motor learning studies indicated positive associations of chronic physical exercise with the initial phase of motor learning, but revealed mixed results for motor retention.

## Discussion

The aim of this systematic review was to investigate the association between chronic physical activity and acute bouts of exercise on motor performance or motor learning in upper extremity motor tasks in older adults. We found no appropriate study examining the influence of an acute bout of exercise on motor performance or motor learning processes in older adults. Therefore, this review focused in the results and discussion sections on the association between chronic physical activity and motor performance and learning. Results of the 25 studies dealing with the influence on motor performance are mixed with a tendency to positive results. Twelve studies showed a positive relationship, eight studies revealed ambiguous results (i.e., some subtasks positive, some no effect) and five did not find effects. For the effect of chronic physical activity on motor learning only three studies were included in the review, showing positive effects on the initial phase of motor learning and ambiguous results with respect to retention.

### Chronic physical activity and motor performance

Differences in study results might depend on the study design, type of exercise, motor task or exercise intensity. Therefore, results will be discussed with regard to these factors.

#### Study design and type of exercise

##### General physical activity behavior as independent variable in cross-sectional studies

The majority (four out of seven) of the cross-sectional studies that investigated the association of chronic physical activity and motor performance by use of subjective measures found no exercise effect [37*, 73*, 80*, 86*]. Only in two cases a positive association between physical activity level and performance in a fine motor task was found [7*, 81*], and one study revealed ambiguous results [1*], depending on the motor task. A comparison between two studies of the same lab, one showing positive and one no associations, indicated that the physical activity level itself might influence results with a higher physical activity score being more beneficial [81*] than a lower one [80*]. Generally, physical activity levels seem to considerably differ between studies depending on the study sample. Thus, one might rate objectively defined physical activity criteria higher than study-specific activity levels, as the latter clearly depends on the specific sample characteristics (cf. below for discussion of median split). Objectively defined activity criteria for older adults are for example at least 150 min of moderate-intensity or 60 min of vigorous-intensity aerobic physical activity per week [49].

One other explanation for the diverging results might be the different measures to assess physical activity, i.e., different physical activity questionnaires. Consequently, studies vary in what they regarded or included as physical activity ([45] for a review ). Furthermore, some studies included in this review did not refer to a standardized physical activity questionnaire or did not state exactly how they acquired their information (e.g., verbally or in written form) [29*, 37*, 54*, 73*, 92*, 94*]. Other studies did refer to a standardized questionnaire, but did not give any information regarding the scores or actual physical activity level of the participants [12*], which also prohibits meaningful conclusions about the participants.

Besides differences in physical activity levels and assessment tools between studies, there is another limitation of physical activity questionnaires. Physical activity questionnaires encompass the physical activity level of the participants subjectively, but are not able to indicate the objective actual physical or cardiovascular fitness level. In this vein, correlations between physical activity calculated by use of a questionnaire and the actual cardiovascular fitness have been shown to be rather weak [62*]. Consequently, it might be more appropriate that future studies assess the actual objective fitness level of the participants to ensure a better validity and enhanced comparability between studies or at least apply standardized physical activity questionnaires. Nevertheless, physical activity questionnaires can give valuable information of the kind of sport participants perform and might therefore provide a valuable additional information (cf., discussion about type of exercise).

##### Cardiovascular fitness level as independent variable in cross-sectional studies

Interestingly, when objective measures were used to assess the cardiovascular fitness level, also no positive effect on motor performance was found in the cross-sectional studies [12*, 19*, 67*]. Two of these studies calculated a median split in order to classify participants as fit and unfit [19*, 67*]. Similar to differences in physical activity levels between studies, a median split might not reflect the “real” cardiovascular fitness level as the value is determined by characteristics of the specific sample. Therefore, depending on the sample, the median split might not differentiate well between the high and low fit participants as it might happened that only moderate fit persons have been recruited and it also might overestimate the fitness level of high-fit participants [41]. Future studies should use objective criteria to judge participants as high or low fit.

##### Targeted cardiovascular exercise as independent variable in interventional studies

Besides these shortcomings in the determination of the physical activity or cardiovascular fitness level, cross-sectional studies cannot provide causal evidence for activity-induced motor performance changes as they were executed in a correlational design. Interventional studies have the advantage that the effectiveness or effects of a targeted exercise can be evaluated and that they provide evidence of causality. Both cardiovascular exercise intervention studies observed enhanced motor performance in upper extremity tasks [3*, 95*]. These findings were additionally supported by the cardiovascular exercise group (i.e., swimming) of the study by Puggaard et al. [56*], which also revealed improved motor performance. Effects have been discussed to rely on improved information processing in corresponding neural circuits like the visuospatial system [3*]. Interestingly, the length of the intervention did not influence study results, which might support the assumption that certain physiological adaptations occur already after several weeks of exercise.

##### Different types of cardiovascular exercise as independent variable in cross-sectional studies

Further, it was discussed whether the type of cardiovascular activity influenced results differently. Dascal and Teixeira [12*] reported no difference in several motor tasks between older runners or general exercisers. However, Hsu et al. [29*] revealed that swimmers performed better in a hand-arm movement task than active control participants. The authors speculated that the same pathways, i.e., from sensory receptors to the cerebellum or other motor centers in the brain and back to motor neurons, are used during swimming as well as motor tasks, so that repetitive (swim)training can improve motor performance [29*]. Whether these ambiguous results are due to the cross-sectional design and the different comparison groups (general exercisers versus active control) or hint to superior effects of different kinds of sports remains highly speculative.

##### Motor-coordination exercises as independent variable in cross-sectional studies

Two (out of five) of the cross-sectional studies, that investigated differences in upper extremity motor performance between persons engaged in motor-coordinative exercises, such as dancing or martial arts, versus general physically active persons were successful to reveal differences [54*, 82*], one showed ambiguous results (depending on the group comparison: [92*]) and two did not find differences between groups [12*, 37*]. Therefore, it seems difficult to judge whether motor-coordinative exercises are more effective than being physically active in general. When comparing persons performing motor-coordinative exercises to inactive participants, two of three further cross-sectional studies revealed that dancers showed better motor performance in a hand-arm fine motor battery ([34*] (marginal significant); [35*]), supporting the positive influence of motor coordinative exercises. However, the third study comparing motor-coordinative exercises to inactive participants by Krampe et al. [37*] did not support this finding, as they revealed no significant difference between martial arts practitioners and inactive controls in force matching or speed tapping tasks, which might indicate that effects of motor-coordinative exercises are task-specific.

##### Motor-coordination exercises as independent variable in interventional studies

Again, interventional studies also with respect to motor-coordinative exercises revealed promising results. This was true for five out of six interventions on multi-component training (intervention group 1: [56*]), tai chi [93*, 94*] and dancing ([33*]; intervention group 3: [56*]). Improved concentration of the participants was discussed as possible explanation for better tai chi ﻿performance [93*, 94*]. A missing effect of tai chi practice on arm movement speed was explained by the fact that tai chi contains slow motion exercises only [93*]. Enhanced performance of dancers compared to non-dancers or increases through a dancing intervention might be attributed to sustained muscle strength, better sensorimotor coordination and better concentration [33*].

Nevertheless, one study conducting a one year multi-component exercise program revealed no effect on motor performance [66*]. However, exercises were performed with a very low intensity and only while sitting or standing and the study did not contain a control group [66*]. It might be speculated, that the intervention counteracted an age-related decline in motor performance, which might have occurred within one year. In contrast, in the second study containing a multi-component exercise intervention (intervention group 1), participants improved their performance in a unimanual coordination task [56*]. Here exercise intensity was 65% of the maximal heart rate [56*]. This suggested that exercise intensity influences results, with higher intensity leading to bigger effects. These findings fit the results of studies from cognitive literature, revealing that high intensity exercise led to increased learning rates in a memory task [91].

##### Strength training as independent variable in interventional studies

Besides coordinative exercise also strength training seems to be an important approach to benefit upper extremity performance. The influencing mechanism, however, seem to be different: the positive associations of both intervention studies on strength training [36*, 52*] might be reasoned by improved finger strength, as enhanced finger strength values correlated with better motor coordination [40]. Thus, in the strength training studies peripheral changes (i.e., intramuscular coordination or hypertrophy) – rather than central adaptations – might be the explaining factor for enhanced upper extremity motor performance.

To summarize, results with respect to type of exercise are heterogeneous, but certain key characteristics seem to influence the results: (1) Effects on motor performance were more pronounced in interventional than in cross-sectional designs regardless whether cardiovascular exercise/fitness or different types of sports activities were investigated. (2) Effects with respect to the type of exercise (i.e., cardiovascular and coordinative exercises) were heterogeneous in cross-sectional studies and seem to not play a role in intervention studies (only one study available). (3) In successful intervention studies, motor performance increased without an improvement in the cardiovascular fitness level, regardless whether the interventions focused on cardiovascular exercise or motor coordination exercises. And, (4) effects seem to be more pronounced in interventions of higher than low intensity.

#### Type of motor task

Although we tried to categorize upper extremity motor performance tasks in reasonable groups, due to the very heterogeneous results it seems difficult to derive a systematic conclusion. It appears that force matching tasks revealed very inconsistent results, manual dexterity and speed tapping task the less promising and hand-arm movements the most promising results. Several explanations might elucidate these findings. In most manual dexterity tasks, motor performance is quantified by the amount of pins plugged into a target within a given time. The range of possible scores is relatively small and the outcome parameters very imprecise, which might hinder to detect differences in performance. Finger tapping tasks (used to assess psychomotor speed) require a central motor program to organize integration of required muscles [73*]. As compared to other motor tasks, this motor program allows only a small amount of degrees of freedom during motor performance. In comparison to all other categories of motor tasks, hand-arm movements seem to be the most complex motor tasks as their performance requires/allows more degrees of freedom. This might indicate that a high physical activity level, cardiovascular fitness level or participation in coordinative sports is positively associated to motor performance in complex motor tasks (i.e., here: hand-arm movement tasks).

#### Other influencing factors and limitations

Besides the study design, type of exercise and motor outcome other influencing factors, e.g., age or gender, might be discussed. Only one study included older adults of different age groups (60–69 years of age and 70–79 years of age; [73*]), but found no significant effect regarding the physical activity and motor performance association between these age groups. The only study included in this review investigating the influence of gender reported also no effect [56*]. However, it is not known how reliable this non-significant gender effect is.

Some studies had very small sample sizes (e.g., [3*]: *n* = 5 per group; [36*]: *n* = 7 in intervention group and *n* = 4 in control group). With these small group sizes, it is hard to derive general assumptions. Another limitation might be the design of the intervention studies. One study included an inactive control group [33*], i.e., the group did not receive any treatment during the intervention period. This could increase the likelihood of a placebo effect, as participants might be aware of general positive effects of physical activity and therefore, be more motivated during posttests than the inactive control participants, which – in turn – might have influenced the results. Nevertheless, even an active control group might not be sufficient to discard placebo effects in interventional studies as participants’ expectations regarding the intervention might already influence study results [6].

### Chronic physical activity and motor learning

“There is a general lack of studies examining the effects of long-term exercise on motor learning and performance so that this area of research must be considered as largely underexplored to date” ([78], p. 9). This statement by Taubert et al. [78] is confirmed by the search results of our systematic review on older adults. Only three studies dealing with the association of chronic physical activity and motor learning were identified. All three studies reported a positive influence of cardiovascular fitness level [19*, 21*] or physical activity level [89*] on the initial phase of motor learning. Therefore, one might conclude that chronic physical activity facilitates initial motor learning. This fits to the proposed idea: because of the fact that the initial phase of motor learning is highly cognitively driven [24] and the well-established positive association of enhanced cardiovascular fitness and cognitive processing in older adults [11], initial motor learning might also be improved by chronic physical activity in older adults.

Motor learning is discussed to be facilitated by neuroplasticity mechanisms as enhanced levels of lactate, BDNF or vascular-endothelial growth factor (VEGF) [78], increases in oxygen transport to the brain [15] or increases in norepinephrine [55]. Consequently, it might be assumed that exercise does not only facilitate learning but also the ability of the aging brain to undergo neuroplasticity This assumption is however, highly speculative and needs to be investigated in future studies. Two of the motor learning studies assessed the neurophysiological correlates of motor performance and motor learning by use of electroencephalography (EEG) alpha activity with contradicting results [19*, 21*]. Etnier & Landers [19*] found a Fitness × Group × Site × Hemisphere interaction. Etnier et al. [21*] did not find an effect of cardiovascular fitness on alpha activity. More recent research revealed that activity in the beta frequency band was associated with motor performance and changes in corticospinal output ([18] for a review). Therefore, one might speculate that analyses of other EEG frequency bands might provide more promising results. Nevertheless, for the association between physical activity and cognitive performance neuroimaging research methods delivered valuable elucidation ([85] for a review). These methods should be (further) used to investigate underlying neurophysiological mechanisms of the association between physical activity and motor performance.

The two studies examining the effects of cardiovascular fitness level on motor learning during delayed retention tests revealed ambiguous results [19*, 21*]. Etnier and Landers [19*] argued for their non-significant results that possible effects might be extenuated, because they chose a submaximal spiroergometry protocol to assess cardiovascular fitness level. This argument might be reasonable, as Etnier et al. [21*], using a maximal cardiovascular capacity protocol, found a significant effect of cardiovascular fitness on retention. Etnier et al. [21*] referred to the same possible mechanisms as already summarized for general effects on motor learning [15, 55].

Besides studies reported in our systematic review, the few studies examining the influence of chronic physical activity on motor learning in groups of young adults or patients reveal noteworthy and auspicious results. One study with young adults revealed increased motor learning after an eight-week cardiovascular exercise program as compared to a control group conducting stretching exercises [58]. Further promising results of exercise induced neuroplasticity, which might improve motor learning, are reported for patients with Parkinson’s disease [27] or are discussed with respect to poststroke rehabilitation [42]. These positive findings regarding chronic physical activity and motor learning might reflect promising approaches for older adults – healthy or different patients groups – as well.

In addition to exercise and physical activity, non-invasive brain stimulation as transcranial direct current stimulation (tDCS) and transcranial magnetic stimulation (TMS) are prominent methods used to improve motor performance and motor learning in healthy individuals as well as in clinical settings ([77] for a review). In this regard, tDCS revealed beneficial results also for older adults ([76] for a systematic review and meta-analysis). However, in the only study with older adults, TMS failed to induce enhanced motor learning [5]. As tDCS and TMS are accompanied with some restrictions (as low reproducibility for tDCS results or medical supervision for TMS), exercise might be a promising and easy applicable approach that might be used in addition or instead of tDCS and TMS.

### Outlook: Acute exercise in older adults

Within our systematic review, we found no appropriate study that examined the influence of an acute bout of exercise on upper extremity motor performance or motor learning processes in older adults. However, it should be mentioned that acute exercise studies with young healthy adults provide promising results, as indicated by enhanced motor learning [61, 74] or increased neuroplasticity ([70] for a review) in the (primary) motor cortex following acute bouts of exercise of different intensities. On the contrary, some studies examined the effect of acute exercise on lower extremity control, i.e., postural stability, in older adults. Postural stability was reduced immediately after exhausting strength-training [46], moderate intensity rehabilitation exercises [16], or moderate intensity cycling [75], implicating that older adults might need to pay increased attention on their balance or might need assistance immediately after physical exercise. Nevertheless, others reported no influence of moderate intensity rehabilitation exercises on postural stability neither in young nor in older adults [17], suggesting that more research with older adults is needed not only in upper, but also lower extremity control. We aim to investigate the association of acute exercise and upper extremity control in older adults in a future study.

### Future directions

Based on our systematic review we suggest to derive the following recommendations for prospective research. To disentangle physical activity, fitness and type of exercise effects, future studies should use an interventional design, apply objective measures of cardiovascular fitness, use objective fitness criteria for group allocations, recruit extreme groups of low-fit and high-fit participants and/or use cardiovascular fitness as continuous variable. Furthermore, studies assessing the influence of particular kinds of sport, regardless whether in a cross-sectional or interventional design, should evaluate the sport participation in more detail. This might provide valid statements about the influence of the particular kind of sport on motor performance with respect to a possible influence of amount and length of participation and – very important – exercise intensity. Additionally, more studies should include neurophysiological and biochemical measures to get further clarification about underlying mechanisms.

## Conclusion

This systematic review reveals heterogeneous findings regarding the association between chronic physical activity and motor performance as well as chronic physical activity and motor learning in upper extremity tasks in older adults. The association between physical activity and motor performance seems to depend on the study design (interventional studies lead to more stable positive results than cross-sectional studies), motor task (complex motor tasks benefit to a higher extend) as well as exercise intensity (stronger effects in interventions with a higher exercise intensity), and seems to be independent from the pure cardiovascular fitness level. A high physical activity or cardiovascular fitness level seem to boost initial motor learning**,** which might be attributed to enhanced cognitive processing of active/fit older adults. Several non-significant findings of cross-sectional studies might be reasoned by the study design (e.g., subjective measures of physical activity or calculation of a median split). Nevertheless, more research is needed to deduce well-founded exercise recommendations leading to improvements in motor performance and boost motor learning processes in older adults.
